# Rosen’s (M,R) system in process algebra

**DOI:** 10.1186/1752-0509-7-128

**Published:** 2013-11-17

**Authors:** Derek Gatherer, Vashti Galpin

**Affiliations:** 1 MRC-University of Glasgow Centre for Virus Research, 8 Church Street, Glasgow G11 5JR, UK; 2 Laboratory for Foundations of Computer Science, School of Informatics, University of Edinburgh, Informatics Forum, 10 Crichton Street, Edinburgh EH8 9AB, UK; 3 Present address: Biomedical & Life Sciences, Lancaster University, Lancaster LA1 4YQ, UK

**Keywords:** Robert Rosen, (*M,R*), Metabolism-replacement, Metabolism-repair, Relational biology, Process algebra, Bio-PEPA, Computability, Turing machine

## Abstract

**Background:**

Robert Rosen’s Metabolism-Replacement, or (*M,R*), system can be represented as a compact network structure with a single source and three products derived from that source in three consecutive reactions. (*M,R*) has been claimed to be non-reducible to its components and algorithmically non-computable, in the sense of not being evaluable as a function by a Turing machine. If (*M,R*)-like structures are present in real biological networks, this suggests that many biological networks will be non-computable, with implications for those branches of systems biology that rely on *in silico* modelling for predictive purposes.

**Results:**

We instantiate (*M,R*) using the process algebra Bio-PEPA, and discuss the extent to which our model represents a true realization of (*M,R*). We observe that under some starting conditions and parameter values, stable states can be achieved. Although formal demonstration of algorithmic computability remains elusive for (*M,R*), we discuss the extent to which our Bio-PEPA representation of (*M,R*) allows us to sidestep Rosen’s fundamental objections to computational systems biology.

**Conclusions:**

We argue that the behaviour of (M,R) in Bio-PEPA shows life-like properties.

## Background

Relational biology is a discipline founded by Robert Rosen (1934–1998) [1-3], based on the previous work of Nicolas Rashevsky (1899–1972), which considers biological network structures using the mathematical tools of category theory. Relational biology might be considered a branch of systems biology, in that it studies the same objects as systems biology, and for similar reasons, namely to improve our understanding of how complex biological processes work. However, its methods and conclusions are so radically different to those of conventional systems biology that one might almost say it constitutes an alternative discourse on the subject. Systems biologists attempt to represent biological network systems as software objects for simulation on computers. These network structures may vary in their degree of complexity, but the difference between simple and complex networks is treated as one of degree rather than of kind. By contrast, relational biologists *define* complex systems as only those systems having *impredicative (self-referencing) components,* and claim that these systems are *non-computable* as functions by Turing machines*.* For relational biologists, complex systems are therefore qualitatively different to simple ones.

A Turing machine is a formal mathematical model of computation, which has a notion of its internal state, an unbounded tape and a read-write head for that tape [4]. It also has a table to determine the next internal state, the symbol to be written to the tape and the direction in which to move the head, all of which depend on the current internal state and the current symbol. The concept of the unbounded tape may be taken to imply limitless time and space for computation. A function is (Turing-) computable if it can be evaluated by a Turing machine which, when given an input to the function, eventually writes the value of the function for that input on its output tape, assuming that the function is defined for the input. No time bound can be imposed on how long the Turing machine will take to output the value.

**Figure 1:**
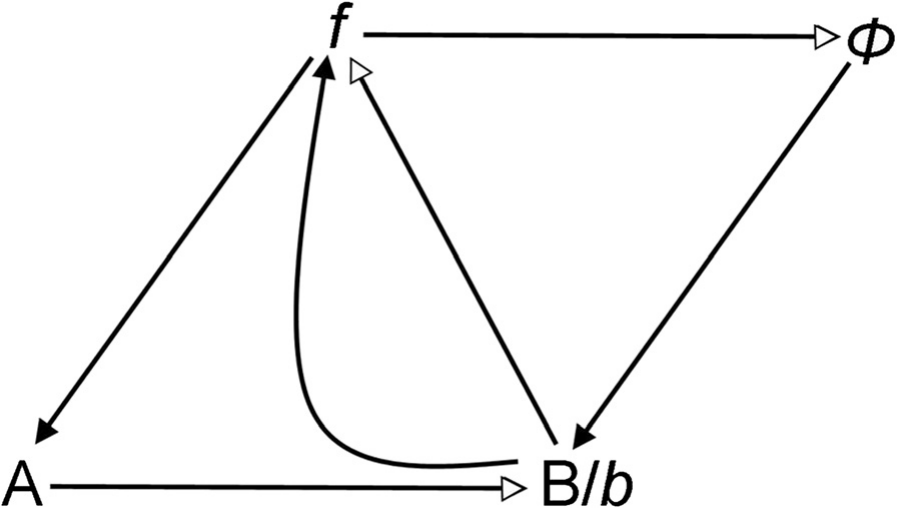
**The Louie-Kercel representation of (*M,R*)**[10]**.** Lines with open arrowheads are metabolic productive reactions, for instance A produces B. Lines with filled arrowheads represent catalysis. Conventionally these point to the substrate of a metabolic reaction. For instance *f* catalyses the production of B from A, and this is indicated by *f* pointing to A. Likewise, Φ catalyses the production of *f* from B, so an open-headed arrow goes from B to *f*, and a filled arrow from Φ to B.

**Figure 2:**
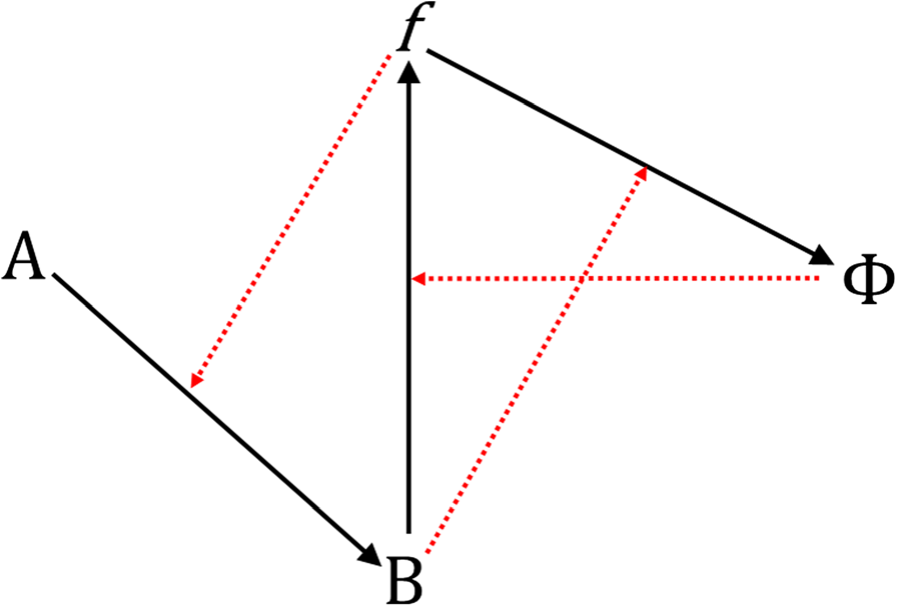
**The Goudsmit representation of (*M,R*)**[15]**.** Black lines are metabolic productive reactions. Red dotted lines represent catalysis – for instance *f* catalyses the production of B from A. Compare with Figure 1, to which this figure is equivalent.

An adequate exposition of the logic of relational biology is impossible within the limits of a research paper. The strategy adopted here is therefore to concentrate on Rosen’s Metabolism-Repair/Replacement system, conventionally abbreviated in brackets as (*M,R*), a very small, but nevertheless allegedly non-computable, network which is the central object of study in modern relational biology. Metabolism-Repair was Rosen’s own phrase, but Metabolism-Replacement is used here for reasons given by previous authors [5]. Readers interested in the construction of (*M,R*), and in particular the reasons why it is deemed to be non-computable, are referred to Louie’s recent book [6], and for contextual material to Rosen’s previous book [7] and the recent series of reviews [5,8-11]. Only the briefest description of (*M,R*) is given in the following paragraphs, referring to Figures 1 and 2, which illustrate (*M,R*) in two ways.

Relational biologists argue that the non-computability of *(M,R)* by Turing machines is achieved through a circular pathway of causal relations, termed *closure to efficient causation*[7]. Furthermore relational biologists seek to demonstrate that such non-computable systems, when represented as graphs, may be surprisingly small in terms of their total number of nodes and edges. Relational biologists do not deny that complex systems can be simulated, but maintain that they can never truly be computed as functions [6,7,12]. Since even the most advanced computers still use Turing architecture (i.e. they are random access stored program machines that are almost Turing machine equivalent), the full computational analysis of complex biological systems is therefore postulated to be beyond our current computing abilities.

Relational biology has therefore concentrated its efforts into pure mathematics to the exclusion of computational analysis. Rosen once quipped that his mentor, Rashevsky, had lived in a time when computers were not easily accessible but, even if they had been, he would not have used them [12]. The abstract graph structures developed by relational biologists are referred to as *models*, and a *modelling relation* is achieved between a system in the natural world and a model in the mathematical world when their *entailment structures*, meaning the totality of their internal causal relations, are identical. *Entailment* is an important concept in relational biology. When a certain pattern of system states necessarily results in a certain outcome, it is said that this outcome is *entailed by* that cause. Relational biologists describe logical entailments in their mathematical work that represent entailments from the physical world and insist, for a correct modelling relation to be obtained, that these entailments must occur in the model. A *simulation*, by contrast, represents the world in a much more approximate way than a model, without the requirement for congruence of internal causal relations. A simulation may be an excellent predictive tool for the natural world, but when it goes wrong one may have no idea why, since its entailment structure is largely a work of creative approximation. Relational biologists maintain that much of conventional systems biology is merely about the development of such approximate simulations [6,7,12].

Rosen also argued that a corollary of the non-computability of complex systems is the fact that they are also non-reducible to their component reactions, in that a model of a complex system cannot be constructed simply by an additive assembly of smaller models of that system’s individual reaction components [7]. By contrast, whatever claims that systems biologists may make about their own anti-reductionism [13], the construction of software representations of biological networks requires the individual representation of component reactions in code, in effect a reduction at the software level. Relational biologists acknowledge that there are many systems that are reducible and algorithmically computable in this way, but none of these are truly complex, and therefore few are really interesting from a biological point of view. A further point of disagreement concerns the issue of mechanism. Systems biologists, and molecular biologists in general, view biological processes as machine-like. Relational biologists maintain that *closure to efficient causation* (i.e. the presence of closed causative loops, as in *(M,R)*) produces an entailment structure, meaning a pattern of necessary causal relationships, that is quite unlike that of a machine. In summary, relational biologists see complex biological systems as non-mechanistic, non-reducible and non-computable [7,12]. Relational biologists are therefore drawn to the conclusion that much of systems biology is at best over-ambitious, an attempt to compute the impossible and to force biological systems into inappropriate mechanist and reductionist straitjackets, and as a result of this that systems biology has no predictive power. On the other hand, systems biologists rarely stay the course with the two book-length expositions of relational biology [6,7], and may even believe that anything is computable, given sufficient time and processing power, despite the fact that computer scientists have long known this to be trivially false [14]. The two disciplines exist on either side of a looking-glass. In this paper, we attempt, like systems biology versions of Alice, to cross the looking-glass and force the tools of one world onto the materials of the other. A certain amount of abrasion inevitably results, both to tools and materials, but we aim to demonstrate that a minimal degree of adjustment is required to achieve congruence between the two approaches. Whether this represents genuine progress or yet another fudge perpetrated by systems biologists, is left to the judgement of the reader.

We now turn to consideration of *(M,R)* in more detail. Figure 1 shows the current standard representation of (*M,R*) as given by Louie & Kercel [10]. Figure 2 shows an alternative provided by Goudsmit [8,15]. Figure 1 follows the representative conventions of relational biology, as follows: B is produced from A, and this is represented by an open-headed arrow from A to B. This productive reaction is catalysed by *f*, and this is represented by a filled arrow from *f* to A. These diagrams can be re-expressed algebraically in category theory within which it is possible to manipulate set theoretical expressions to deduce that (*M,R*) contains impredicative set structures, i.e. sets that are members of themselves [10]. These form the centrepiece of the conclusion that (*M,R*) as a function is not Turing-computable. Some authors have questioned the mathematical correctness or completeness of the various category theory manipulations performed by relational biologists [16-18]. Others have disputed these claims [19-22]. The present paper concentrates solely on the diagrammatic representation of (*M,R*) and attempts to re-express it in process algebra.

Rosen intended (*M,R*) to be interpreted very generally, as a representation of a whole metabolic system B produced from an entire set of nutrient sources A. Metabolic activity in B is catalysed by a set of enzymatic functions *f* and maintained by replacement functions Φ. Here, in the spirit of the previous simulation by Prideaux [23], we consider (*M,R*) as a representation of a single metabolic pathway, with A, B, *f* and Φ as individual entities, rather than the sets of metabolites and enzymes implied in Rosen’s use of *(M,R*) as a general schema. The Goudsmit representation (Figure 2) can be thought of as emphasizing this narrower interpretation, although it was not necessarily intended as such by its author. This therefore makes (*M,R*) more readily comprehensible to systems biologists, in effect bringing (*M,R*) as close as possible to the kind of network diagram typically displayed in the systems biology literature. In the Aristotelian language used by relational biologists, A is the material cause of B, demonstrated in Figure 1 by the open arrowhead and *f* is the effective cause of B, represented by the closed arrowhead ending on the material cause, A. The combination of two sequential closed and open ended arrows together represents entailment – the presence of *f* entails (i.e. necessarily results in) the presence of B. A similar relationship of material causation exists between B and *f*, this time effected by Φ, and between *f* and Φ, effected by B, which is represented as *b* when acting as an effective cause. This gives rise to a circular entailment structure: *f* entails B which (as *b*) entails Φ which entails *f* and so on. Such a circular entailment structure results in self-reference or impredicativity and therefore, it is claimed, non-computability by a Turing machine.

Figure 2 represents (*M,R*) in narrower terms, A is a substrate for the production of B under the catalytic action of the enzyme *f*. B is also an enzyme (represented as *b*) which catalyses the production of Φ from *f*. Similarly Φ catalyses the production of *f* from B. Rather than attempting to represent the whole of metabolism and replacement, this second version of (*M,R*) demonstrates circular entailment and impredicativity in a single network with four components. It can thus readily be seen how (*M,R*), as a network motif [24], may repeatedly intrude into larger networks. If (*M,R*)’s non-computability really presents an insuperable obstacle to understanding complex biological systems, then by implication this raises questions concerning the usefulness of computational systems biology.

Table 1 summarizes previous attempts to simulate (*M,R*). As long ago as 1974, Varela *et al.*[25] simulated an autopoietic system as a tessellation automaton on an IBM 360. The relevance of this early result to the computability of (*M,R*) systems only became apparent once Letelier *et al.*[35] demonstrated that autopoietic systems constitute a sub-class of (*M,R*) systems, by which time several further simulations of autopoietic systems had been developed [26-31]. Cho *et al.*[32] produced an extension to Rosen’s (*M,R*) system by allowing for mutation, defined simply as alterations to the system while it is in operation, transforming (*M,R*) from “a description in terms of mappings into a dynamical model familiar to control engineers”. This dynamical model was then represented as a hybrid automaton operating with non-deterministic state transitions. This automaton was then applied to a real example, the xanthophyll cycle in plants. Other researchers attempted to construct realistic biological network structures that capture all the required properties of (*M,R*). Cardenas *et al.*[8] demonstrated that both a completely abstract system that merely manipulated numbers and a plausible biochemical network both fulfilled the theoretical requirements of (*M,R*). They then simulated the latter using MatLab, COPASI and MetaTool [33]. A similar philosophy was adopted by Prideaux [23] who constructed an (*M,R*) system using the SPICE circuit simulator software. The present study follows previous studies (Table 1) in attempting to simulate (*M,R*) on a computer. This is the first such attempt using the process algebra Bio-PEPA [36,37].

**Table 1:** **Summary of attempts to adapt ( *M,R *) or its (alleged) derivatives, to run as simulations in computer systems**

**Type of simulation**	**Software system**	**Authors**
Autopoietic	Tesselation automaton	Varela *et al.* (1974) [25]
Autopoietic	SWARM	McMullin & Varela (1997), McMullin (2004) [26,27]
Autopoietic	Assorted others	Zeleny (1978), Breyer *et al.* (1998), Ikegama *et al.* (2002, 2008) [28-31]
Extended (*M,R*)	Hybrid automaton	Cho *et al.* (2005) [32]
Full (*M,R*)-consistent example	MatLab/COPASI/MetaTool	Piedrafita *et al.* (2010, 2012) [33,34]
Full (*M,R*)-consistent example	SPICE	Prideaux (2011) [23]
Compact (*M,R*)	Bio-PEPA	This paper

## Results

**Figure 3:**
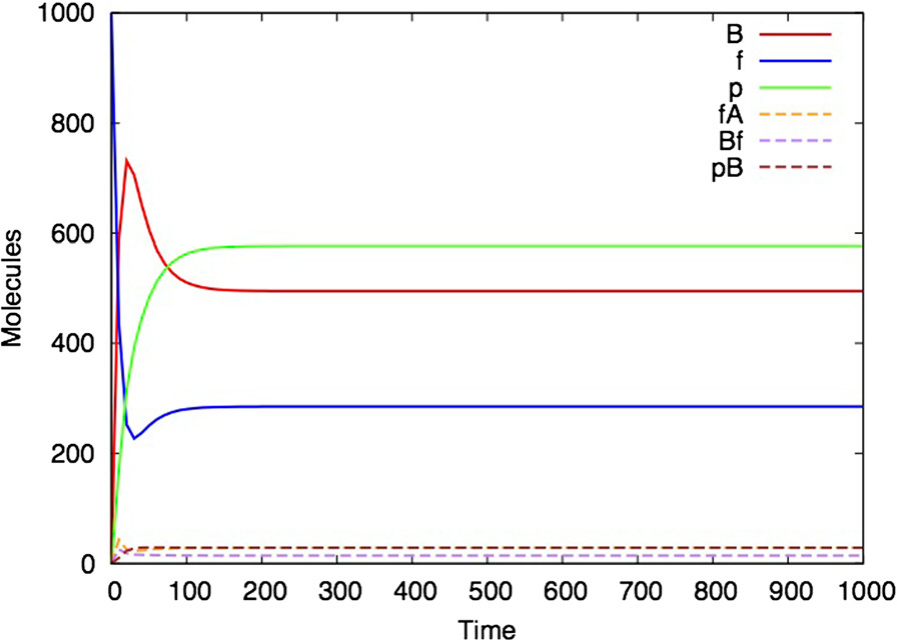
**ODE analysis for the Bio-PEPA *(M,R) *model.** (Parameters: k1 = l1 = m1 = 0.01, k2 = l2 = m2 = 100, k3 = l3 = m3 = 1, d1 = 0, d2 = 0.05, d3 = 0.02426. Initial quantities: A_init = f_init = 1000, B_init = p_init = fA_init = pB_init = Bf_init = 0).

**Figure 4:**
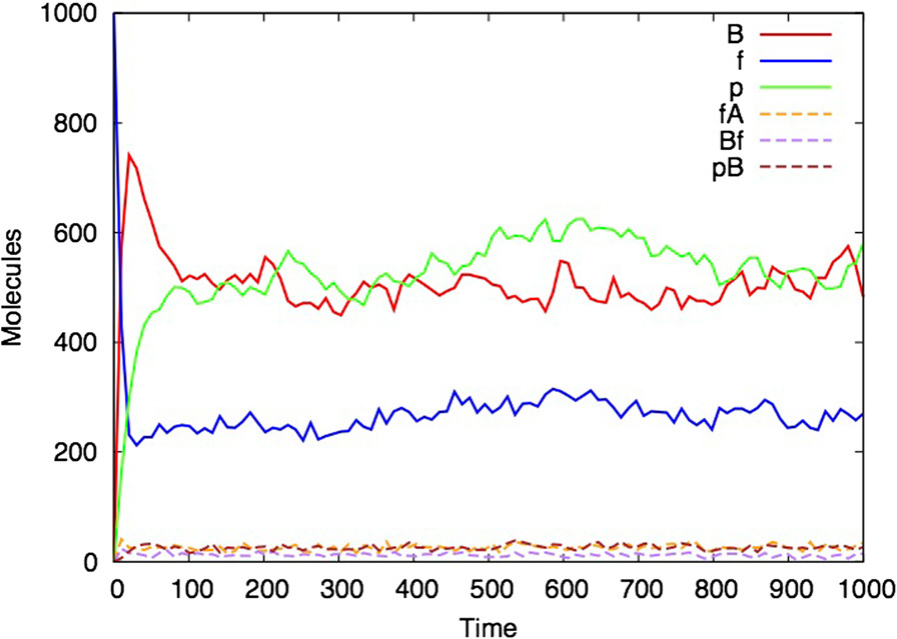
**Single stochastic simulation of the Bio-PEPA *(M,R) *model.** (Parameters and initial values as for Figure 3).

**Figure 5:**
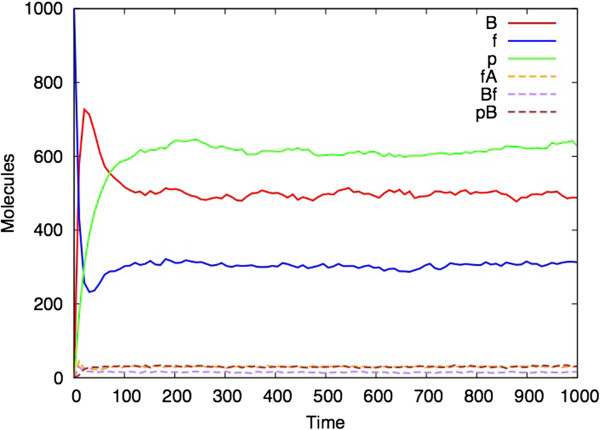
**Average of ten stochastic simulations of the Bio-PEPA *(M,R) *model.** (Parameters and initial values as for Figure 3).

It is immediately evident that the Bio-PEPA *(M,R)* model has dynamic behaviour. Figure 3 shows an ordinary differential equation (ODE) analysis of the model with parameters chosen so that the model converges to steady state behaviour. Since ODEs are a continuous representation of a system that actually consists of discrete components, it is also interesting to perform a stochastic simulation (based on Gillespie’s approach [38]) of the model. Each stochastic simulation represents one possible trajectory of the model (rather than average behaviour which is what the ODE analysis provides, in general). Figure 4 shows a single stochastic simulation and Figure 5 shows the average of ten such simulations, illustrating the smoothing effect of averaging over multiple simulations. At steady state, there are 1000 molecules of A, approximately 577 of p (here we chose to use p instead of Φ), 495 of B, 286 of f, 28 of pB, 28 of fA and 14 of Bf. The ODE analysis can be performed for larger number of molecules by appropriately scaling the rate constants, and an analysis for A with an initial quantity of 10^7^ molecules is given in the Additional file 1.

**Figure 6:**
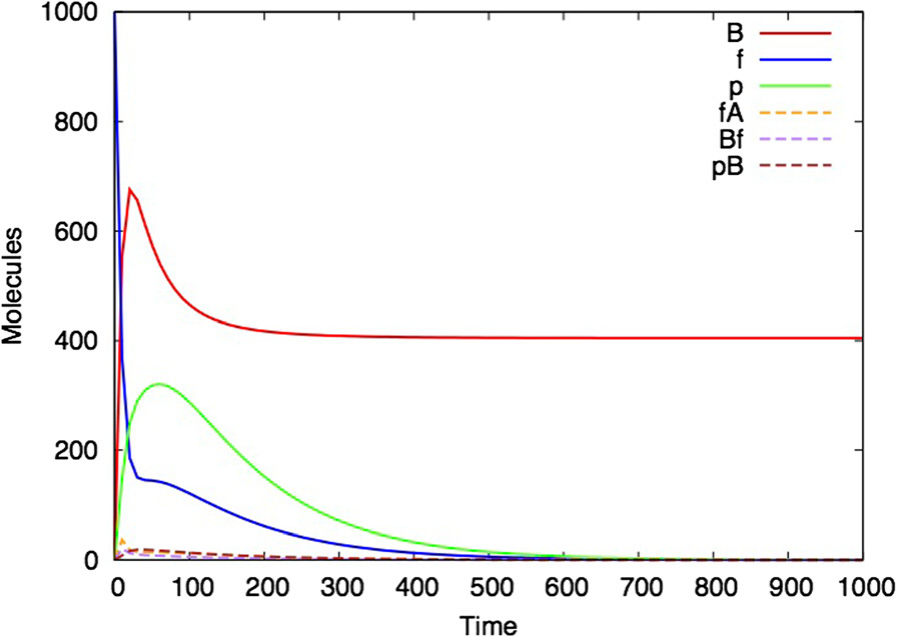
**ODE analysis of the Bio-PEPA *(M,R) *model.** (Parameters and initial values as for Figure 3 except d2 = 0.07).

By contrast to Figures 3, 4 and 5, Figure 6 shows analysis of the model with one parameter changed: d2 = 0.07 instead of d2 = 0.05 in the proceeding figures. This bistability in (*M,R*) simulations has also been found by Piedrafita *et al.*[34]

**Figure 7:**
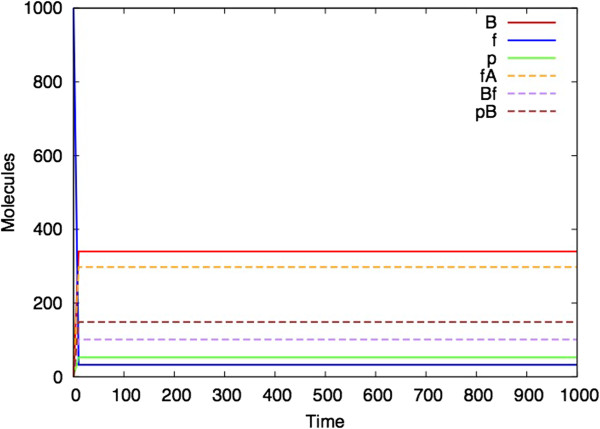
**ODE analysis of the Bio-PEPA *(M,R) *model.** (Parameters: k1 = l1 = m1 = 1, k2 = l2 = m2 = 100, k3 = m3 = 10, l3 = 20, d1 = 0, d2 = 60, d3 = 19.2666666. Initial quantities: A_init = f_init = 1000, B_init = p_init = fA_init = pB_init = Bf_init = 0).

**Figure 8:**
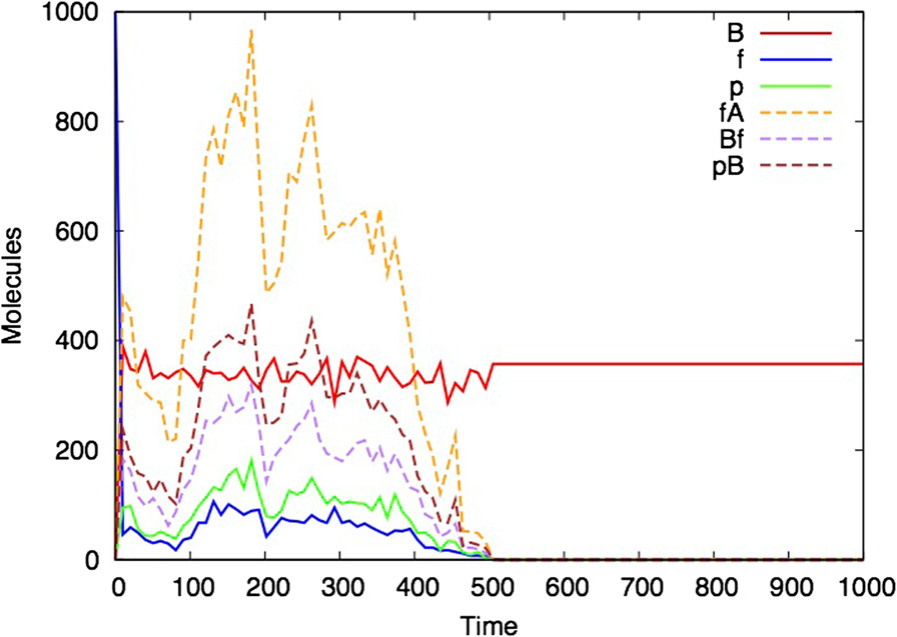
**Single stochastic simulation of the Bio-PEPA *(M,R) *model.** (Parameters and initial values as for Figure 7).

It can also be the case that for a given set of parameters, different stochastic simulations can have different outcomes (even if the ODE analysis shows a steady state). The model in Figures 7 and 8 uses different parameters to those illustrated in Figures 3, 4, 5 and 6. Figure 7 shows the ODE analysis with a steady state outcome and Figure 8 shows a stochastic simulation with the same parameters and initial starting conditions that nevertheless ‘dies’ at time 500 after which no more reactions occur.

The explanation for the fact that the parameter regime in Figures 7 and 8 is more likely to produce a simulation with a finite lifespan, whereas the parameter regime shown in Figures 3, 4, 5, 6 and 7 more consistently survives beyond the 1000 time unit point, appears to be related to the steady state values of B, f and p that are obtained in each simulation. In the former case, these values are closer to zero than in the latter case, and hence it is more likely for two of these species to reach zero at the same time which brings all reactions to a halt.

We have also modelled the STU model presented in Piedrafita *et al.*[33] in Bio-PEPA. Our Bio-PEPA model agrees with the ODE analysis presented in that paper and also allows us to do stochastic simulation. The results of the simulation (not shown) demonstrate that when STU is modelled stochastically, it is a possibility that all reactions cease close to the initial time when molecule counts are low but that if the species can be established in sufficient numbers then the reactions are likely to continue indefinitely.

## Discussion

Rosen’s (*M,R*) system has been encoded in the Bio-PEPA process algebra and this representation has been simulated in the Bio-PEPA Java Eclipse tool. In some runs, the system achieves a steady state and in others it exhibits initial activity and then becomes quiescent, similar to results from other simulations [33]. Due to the use of a stochastic sampling algorithm, identical input parameters can occasionally produce either “life” or “death”. (*M,R*) is therefore shown to be a fragile system under certain parameters and starting conditions, vulnerable to small perturbations, but nevertheless one that can on occasions achieve a metabolic equilibrium. Study of the behaviour of the model under an exhaustive range of input parameters, has, however, not been performed. It is possible that the system is more fragile in certain input ranges than others, but that remains unknown.

This paper represents the latest in a handful of attempts to produce realizations of (*M,R*) in computers (Table 1). It differs from previous versions in that it uses as its starting point the diagrammatic representation of (*M,R*) (Figure 1), rather than any of the mathematical formulations. Our simulation represents a hypothetical universe where biological networks are structured precisely as described by (*M,R*). What therefore are its implications for Rosen’s thesis that (*M,R*) network structures are non-computable? Given the demonstrated activity in Figures 3, 4, 5, 6, 7 and 8, one might jump to the conclusion that Rosen’s theory has been falsified, but for several subtle reasons it is not possible to state this without reservations.

Previous attempts to re-express (*M,R*) in mathematical terms that might be more conducive to construction of a Turing computable model have often met with the response that they misrepresent in some way the structure of (*M,R*). For instance, Goertzel’s transformation of (*M,R*) in category theory into a representation in division algebra [39] was dismissed by Louie for the reason that it altered the entailment structure [21]. Similarly, the re-expression of (*M,R*) in terms of λ-calculus by Mossio and colleagues [40] omitted a crucial component [8]. The latter is of particular significance since anything that can be represented in terms of λ-calculus can be encoded in the computer language LISP, and is therefore by implication Turing-computable. This insistence that no alternative mathematical expression of (*M,R*) has correctly captured the entailment structures has also been extended to practical attempts to simulate (*M,R*) on computers. For instance, Louie takes this stance [22] relative to the work of Prideaux [23,41], insisting it is merely a simulation of (*M,R*) and not a successful computation of the model. This issue of simulation versus modelling has also been a subject of much discussion in the field of autopoietic systems. A case has been made that autopoietic systems are subsets of (*M,R*) [35]. Within the autopoietic field the issue of Turing computability has been much debated [25-31,42]. Those persuaded of the computability of autopoietic systems have therefore deduced that (*M,R*) must also be Turing-computable [43]. Nevertheless, it has also been maintained that autopoietic systems as constructed so far in software are not congruent with (*M,R*) entailment structures [8,35]. The relevance of this debate to the computability or otherwise of (*M,R*) depends entirely on the validity of the parallel drawn between (*M,R*) and autopoietic systems, for which there is still only a single derivation [35].

The model presented here is also open to the same potential criticism: that we have distorted the entailment structure of (*M,R*) or have not completely achieved the requirement for all entailments to be internal to the system. Insofar as our Bio-PEPA realization of *(M,R)* resembles Prideaux’s SPICE realization [23], it is vulnerable to Louie’s same criticism of the latter [22], if that criticism is accepted. Nevertheless, we believe that all the necessary productive, or perhaps one might say the synthetic, entailments represented by arrows in Figures 1 and 2 are captured in our Bio-PEPA representation. Rosen did not specify within the context of (*M,R*) where the raw material for A was to be obtained or how to prevent its dissipation or that of any of the other components. The questions of how to define ancillary system properties, such as the boundaries of the reaction compartment, arise only when one insists on forcing (*M,R*) into a biologically plausible context.

A related issue concerns the way that the entailment structure of (*M,R*) is translated into software processes. Bio-PEPA is used to simulate (*M,R*) in both stochastic and deterministic ways. In the stochastic implementation, the entailment structure is “sampled”, meaning that one randomly chosen entailment will update the whole system state based on the rules of that entailment and the existing system conditions. In the deterministic version, there is no such “sampling”, but we set an arbitrary starting condition which determines the subsequent development of the system. The deterministic version is obviously the one closest to Rosen’s notion of entailment, but given that its behaviour is indistinguishable from the average of hundreds of runs of the stochastic version, we do not believe that this issue is important.

The second potential criticism is that the unfolding of the system through time in both stochastic and deterministic versions of the simulation, is represented by a sequence of states. Rosen was quite explicit that relational systems such as (*M,R*) do not have states, since states are by Rosen’s definition a feature of systems where the entailment comes from outside the system, i.e. systems existing in a changing environment [7]. All computational simulations of (*M,R*) to date are temporal and pass through a sequence of states. At this point, systems biologists may insist that all models must of necessity have states since organisms (like computers) exist in time. However, many of our standard representations of biological networks are state-free, without it being much commented on. For example the diagrams of metabolic cycles found in biochemistry textbooks have no explicit time component. State-free does not therefore necessarily imply atemporality, merely a condition of representation. We consider this issue to be unresolved.

We now turn to issues related to computability, and whether *(M,R)* is noncomputable. The first issue is that of definition of computability [4]. The theory of computation in general, and specifically the definition of computability, applies to partial functions, i.e. those that need only be defined on a proper subset of their input set. The computability of a function does not require termination for an input value on which that function is not defined, so it is not necessary for a Turing machine computing that function to terminate for every input. This issue of partial versus total functions (meaning those defined over all inputs) is raised in the appendix of Mossio *et al*. [40] Cárdenas *et al.*[8] in their rebuttal of that paper, state that Rosen’s definition of computability requires termination after a finite number of steps, presumably regardless of the input. However, as suggested by Mossio *et al.*[40] in their appendix, Rosen’s notion of computability is non-standard since if the function is not defined for an input, the standard definition of computability does not require termination. If Rosen’s definition of computability does not match current mathematical understanding of computation, then it severely weakens his argument about the non-computability of life.

The second item we discuss is impredicativity. One of the foundations of relational biology’s claim that (*M,R*) is not computable is the existence of an impredicative set within its entailment structure when developed in category theory [6,7]. However, impredicativity in and of itself is insufficient to ensure non-computability. For example, recent work in theoretical computer science has produced techniques for rendering impredicative sets (non-well-founded sets, or sets defined co-inductively) as predicative ones [40,44]. This opens the possibility of transforming (*M,R*) in such a way as to eliminate its impredicative sets and show its Turing-computability. Some initial work has been done on applying hypersets, a method for representing impredicative and predicative sets under a single graphical notation, to (*M,R*) [44]. However, this is not fully worked out with respect to (*M,R*) as yet.

Mossio *et al.*[40] expressed the basic *(M,R)* system in λ-calculus, using recursion to capture the impredicativity. They then used a fixed point operator to identify functions for *f*, B and Φ. They emphasise that “…Rosen’s definitional infinite regress is perfectly handled by recursion, in particular as formalized in the λ-calculus” ([40] page 494). Since the expression of a function in the λ-calculus and computability of the function by a Turing machine are equivalent, this means that Mossio *et al.*[40] purport to have shown that a general form of the *(M,R)* system can be expressed as a computable function. However, this has been questioned by Cárdenas *et al.*[8] on the grounds that Mossio *et al.*[40] do not accurately represent *(M,R)*, specifically that they fail to capture the difference between B as product and *b* as catalyst (see Figures 1 and 2). This refers to the original formulation of (*M,R*) by Rosen as a depiction of a whole living system, where B represents the totality of the products of metabolism from nutrient sources represented by A. Only a subset of those products would have a catalytic function, and they are represented as *b*. Since the present adaptation of (*M,R*) represents a single metabolic pathway, B and *b* are the same entity. It should be noted that this is one respect in which our simulation differs from that of Prideaux [23], who treated B and *b* as separate entities and incorporated a conversion parameter between them.

This brings us to the search for such predicted real (*M,R*) systems. Some of the work on simulation of (*M,R*) has involved attempts to define a plausible biological network that satisfies the constraints applied by the model [8,20,33]. However, it is also possible to approach this from the opposite direction. Given that a large amount of data is now available from the systems biology world on the structure of real biological networks, it may be possible to search known networks for topological structures similar to (*M,R*). The simplicity of (*M,R*) interpreted in its micro-form, with four entities and three reactions, suggests that it would be extremely common. Defining (*M,R*) as a network motif and searching for it in databases of networks would appear to be straightforward [24]. However, there is one major obstacle to this, namely that (*M,R*) mixes two kinds of reactions, synthetic and regulatory, that are frequently represented separately. Lerman *et al.*[45] call these M-models and E-models respectively. For instance, classical metabolic diagrams (M-models) frequently feature reaction intermediates and omit information on the catalysts of those reactions. Likewise, genetic regulatory network diagrams (E-models) tend to represent which genes activate or interact with others without necessarily specifying any of the products of those reactions. The search for real examples of (*M,R*) would be best focussed on models combining both metabolic and expression data. Lerman *et al.* have produced such a model, which they call an M-E model, for *Thermotoga maritima*, available in systems biology markup language (SBML) [45]. The resources to answer this question are therefore beginning to become available, and this would seem to be the obvious next step in the integration of relational and systems biology.

## Conclusions

*(M,R)* is presented here in a version using the process algebra Bio-PEPA. This is the latest in a series of attempts to realise *(M,R)* in software form. Under some input parameter configurations, the system can achieve a stable active state, whereas with others it dies. As in real living systems, stochastic factors influence the outcome. This “life-like” property suggests to us that computational relational biology is possible, provided it is recognised that its computational component will consist of open-ended processes.

## Methods

Bio-PEPA is a quantitative process algebra, specifically designed for modelling the interaction of proteins and other molecules [36]. It was developed from the stochastic process algebra PEPA which was introduced for modelling the behaviour and performance of artificial systems such as computer networks [37]. A molecular species S is expressed in Bio-PEPA as follows:

Where a1,…,an are reaction names, k1,…,kn are stoichiometric coefficients and the operators op1,…,opn are each one of the following:

● >> indicates the role of S is as a product,

● << indicates the role of S is as a reactant,

● (+)indicates the role of S is as an activator, or catalyst,

● (−)indicates the role of S is as an inhibitor.

Species are then combined to form a model

where there are p species, S1 through to Sp and their associated molecule counts or concentrations, x1 through to xp.

To make this more concrete, consider the reactions in the (*M,R*) model. It consists of three enzymatic reactions of the form  where *S* is the substrate, *E* is the enzyme and *P* is the product. Considering one of them  (here we chose to use *p* instead of *Φ*) this can be expressed as the three bimolecular reactions

● *B + p → pB* at rate *l*_*1*_

● *pB → p + B* at rate *l*_*2*_

● *pB → p + f* at rate *l*_*3*_

which show how the substrate binds to the enzyme after which the substrate and enzyme unbind or alternatively the product is created and the enzyme is freed. These three species can be expressed in Bio-PEPA using the following text.

Considering reaction r_l3, this shows that both p and f are products of the reaction and pB is the only reactant. In each case the stoichiometric coefficient is one, meaning that only one molecule of each species is involved in the reaction. The rate at which this reaction takes places is specified by

using mass action. Furthermore the rates of the other reactions can be defined by

again using mass action, where l1, l2 and l3 are rate constants. Just considering these four species, the overall model in its initial state could then have the form

where there are B_init molecules of B and p_init molecules of p, and no molecules of either f or pB.

A similar approach is used to express  in Bio-PEPA, and the rate constants used are m1, m2 and m3. The reaction  requires a slight modification since the amount of *A* remains constant as it is provided by the environment. The rate constants for this reaction are k1, k2 and k3. Additionally, each of B, f and p have a degradation term which is also mass action based, so the rate equation consists of a rate constant multiplied by the current quantity of the species. The rate constants are d1, d2 and d3 respectively. The full Bio-PEPA model is contained in the Additional file 1 and the Bio-PEPA Eclipse Plug-in can be downloaded from http://biopepa.org.

The style of definition that Bio-PEPA uses is reagent-centric which means that the reaction capabilities of each species are defined, as opposed to the reaction-centric notation *A → B*. It is easy to switch between these two notations and Additional file 1: Figure S2 provides output from the Bio-PEPA Eclipse Plug-in tool illustrating the reaction-centric description of the Bio-PEPA model. One of the advantages of Bio-PEPA is that a model can be defined once and then different types of analysis can be performed.

## Competing interests

The authors declare that they have no competing interests.

## Authors’ contributions

DG conceived the study, and shared in writing the text. VG performed the Bio-PEPA analysis, and shared in writing the text. All authors read and approved the final manuscript.

## Supplementary Material

Additional file 1: Figure S1 ODE analysis of Bio-PEPA (M,R) model. (Initial values of A and f: 10^7^. Parameters from Figure 3 appropriately scaled). **Figure S2.** Screenshot from the Bio-PEPA Eclipse Plug-in showing the reaction-centric view of the Bio-PEPA *(M,R)* model. Click here for file
