# Supplementary material for: Rosen’s (M,R) system in process algebra
Source: BMC Syst Biol. 2013 Nov 17;7:128. doi: 10.1186/1752-0509-7-128 (PMC3879122; doi:10.1186/1752-0509-7-128)
Supplement: Additional file 1: Figure S1 — ODE analysis of Bio-PEPA (M,R) model. (Initial values of A and f: 107. Parameters from Figure 3 appropriately scaled). Figure S2. Screenshot from the Bio-PEPA Eclipse Plug-in showing the reaction-centric view of the Bio-PEPA (M,R) model. [file 1752-0509-7-128-S1.doc]

**Additional file 1**

//Rosen's (M,R) model using mass action

//Parameters

k1 = 0.0001;

k2 = 0.01;

k3 = 0.001;

l1 = 0.0001;

l2 = 0.01;

l3 = 0.002;

m1 = 0.0001;

m2 = 0.01;

m3 = 0.001;

d1 = 0.0;

d2 = 6.0;

d3 = 1.92666666;

//Initial values

A_init = 1000000;

B_init = 0;

f_init = 1000000;

p_init = 0;

fA_init = 0;

pB_init = 0;

Bf_init = 0;

//Functional Rates

kineticLawOf r_k1 : k1 * f * A;

kineticLawOf r_k2 : k2 * fA;

kineticLawOf r_k3 : k3 * fA;

kineticLawOf r_l1 : l1 * p * B;

kineticLawOf r_l2 : l2 * pB;

kineticLawOf r_l3 : l3 * pB;

kineticLawOf r_m1 : m1 * B * f;

kineticLawOf r_m2 : m2 * Bf;

kineticLawOf r_m3 : m3 * Bf;

kineticLawOf r_d1 : d1 * B;

kineticLawOf r_d2 : d2 * f;

kineticLawOf r_d3 : d3 * p;

//Components

A = (r_k1,1) (+) A;

B = (r_k3,1) >> B +

(r_l1,1) << B + (r_l2,1) >> B +

(r_m1,1) << B + (r_m2,1) >> B + (r_m3,1) >> B + (r_d1,1) << B;

f = (r_k1,1) << f + (r_k2,1) >> f + (r_k3,1) >> f +

(r_l3,1) >> f +

(r_m1,1) << f + (r_m2,1) >> f + (r_d2,1) << f;

p = (r_l1,1) << p + (r_l2,1) >> p + (r_l3,1) >> p +

(r_m3,1) >> p + (r_d3,1) << p;

fA = (r_k1,1) >> fA + (r_k2,1) << fA + (r_k3,1) << fA;

pB = (r_l1,1) >> pB + (r_l2,1) << pB + (r_l3,1) << pB;

Bf = (r_m1,1) >> Bf + (r_m2,1) << Bf + (r_m3,1) << Bf;

//System Model

A[A_init] <*>

fA[fA_init] <*> B[B_init] <*>

pB[pB_init] <*> f[f_init] <*>

Bf[Bf_init] <*> p[p_init]

**
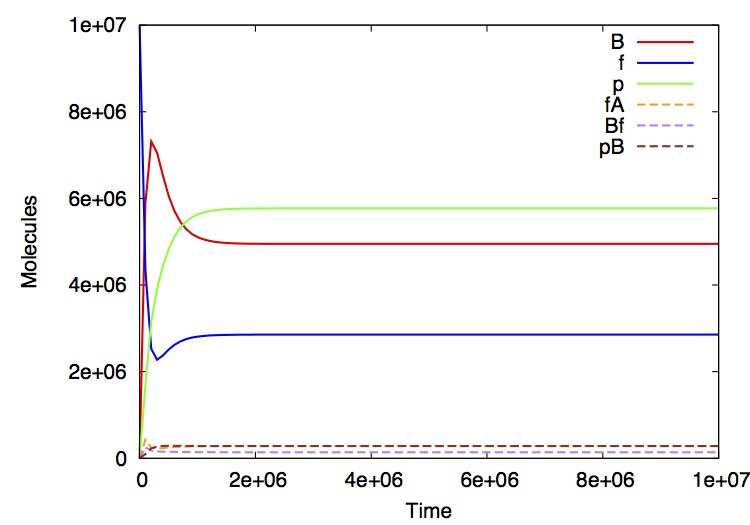
**

**Figure S1**: ODE analysis of Bio-PEPA (M,R) model

(Initial values of A and f: 10^7^. Parameters from Figure 3 appropriately scaled)

**
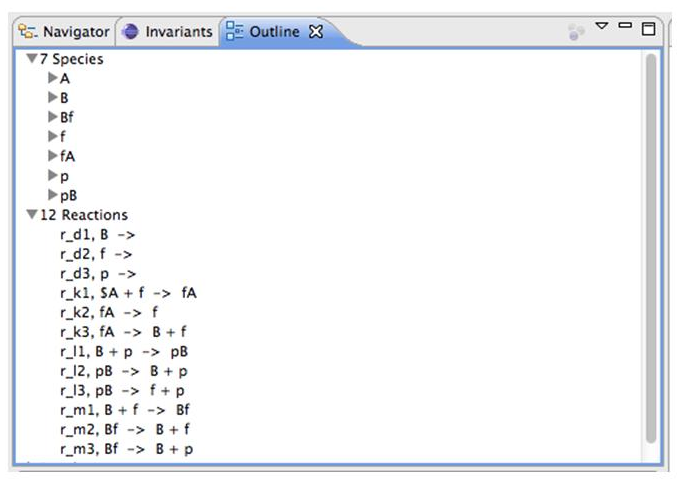
**

**Figure S2**: Screenshot from the Bio-PEPA Eclipse Plug-in showing the reaction-centric view of the Bio-PEPA *(M,R)* model.
